# Use of mixed gas pneumoperitoneum during minimally invasive surgery: a systematic review of human and mouse modelled laparoscopic interventions

**DOI:** 10.1007/s11701-024-01971-1

**Published:** 2024-05-17

**Authors:** Leon Chen, Prokar Dasgupta, Nikhil Vasdev

**Affiliations:** 1 Faculty of Life Sciences & Medicine, King’s College London, London, UK; 2 Department of Urology, Guy’s & St Thomas’ NHS Foundation Trust, London, UK; 3 MRC Centre for Transplantation, King’s College London, London, UK; 4 Hertfordshire and Bedfordshire Urological Cancer Centre, Department of Urology, Lister Hospital, East and North Hertfordshire NHS Trust, Stevenage, UK; 5 School of Life and Medical Sciences, University of Hertfordshire, Hatfield, UK

**Keywords:** Pneumoperitoneum, Laparoscopy, Complications, Adhesions, Mixed gas

## Abstract

The formation of pneumoperitoneum involves the process of inflating the peritoneal cavity during laparoscopic and typically uses CO_2_ as the insufflation gas. This review aims to identify ideal gas mixtures for establishing the pneumoperitoneum with animal and human studies undertaken up to the writing of this review. A systematic search of PubMed, OVID, and clinicaltrials.gov was performed to identify studies on the utilisation of mixed gases in laparoscopic surgery, including non-randomised/randomised trials, animal and human studies, and studies with inflating pressures between 12 and 16 mmHg. ROBINS-I and RoB2 tool was used to assess the risk of bias. A narrative synthesis of results was performed due to the heterogeneity of the studies. 5 studies from the database search and 5 studies from citation search comprising 128 animal subjects and 61 human patients were found. These studies collated results based on adhesion formation (6 studies), pain scores (2 studies) and other outcomes, with results favouring the use of carbon dioxide + 10% nitrous oxide + 4% oxygen. This has shown a significant reduction in adhesion formation, pain scores and inflammation. The use of this gas mixture provides promising results for future practice. Several of the studies available require larger sample sizes to develop a more definitive answer on the effects of different gas mixtures. Furthermore, the number of confounding factors in randomised trials should be reduced so that each component of the current suggested gas mixture can be tested for safety and efficacy.

**Supplementary Information:**

The online version contains supplementary material available at 10.1007/s11701-024-01971-1.

## Introduction

The formation of pneumoperitoneum involves the process of inflating the peritoneal cavity to provide adequate space and visualisation of the operating area. Currently, the formation of pneumoperitoneum is largely done with carbon dioxide (CO_2_) as the insufflation medium. This gas is inexpensive, unreactive, non-explosive and has higher blood solubility than other mediums such as air or oxygen, reducing the risk of embolisms [1], which covers many points for being an ideal gas in establishing the pneumoperitoneum [2]. Despite these characteristics, CO_2_ is not a flawless gas and does have limitations. At the site of pneumoperitoneum, long-term CO_2_ exposure results in low tissue partial pressures and increased inflammation due to peritoneal irritation [3] resulting in increased formation of adhesions; subsequently causing infertility, bowel obstructions [4, 5] and complications during new interventions [6]. Systemically, prolonged CO_2_ pneumoperitoneum results in hypercapnia and acidosis, and is associated with cardiopulmonary complications such as pulmonary oedema, cardiac arrhythmias, and tachycardia [2, 7].

Prior experimentation to find alternatives to CO_2_ include helium (He), nitrous oxide (N_2_O) and oxygen (O_2_), and systematic reviews have covered the clinical outcomes of these studies, [8] with concerns about the safety and efficacy of other single-gas type mediums still apparent. However, no reviews at the time of writing have covered the use of gas mixtures to form the insufflation medium and currently, the use of gas mixtures is under-recognised with most topic-relevant studies centred on animal experimentation; only a handful of human studies have been completed.

We therefore set out to:

- Identify ideal gas mixtures for establishing pneumoperitoneum in abdominal and gynaecological pelvic surgery with current animal and human studies already undertaken (and with results) up to the writing of this review.
- Synthesise results from both non-randomised experimental and randomised control trials (RCT).
- Identify the risk of adhesion formation with each gas mixture medium as the primary outcome.
- Identify other outcome variables that may be affected due to the use of CO_2_ and promote the field of research to identify other suitable gas mixtures for insufflation.

## Materials and methods

This study was conducted utilising the PRISMA guidelines for reporting [9]. A priori protocol was registered on the PROSPERO database with ID CRD42023395598.

### Search strategy

A comprehensive search of PubMed, Medline, EMBASE and clinicaltrials.gov databases was performed using the following search terms, undertaken on 13/12/2022 presented in Table 1. This search included a combination of keywords and MeSH terms for laparoscopic surgery and gas mixture utilisation on PubMed and MEDLINE databases and Boolean operators such as ‘AND’ and ‘OR’ were utilised. Additional articles were identified through references.

**Table 1:** Shows the search terms utilised and the Boolean operators utilised for each database used

Database	Search terms	Filters	Total searches
PUBMED	((Pneumoperitoneum[Title]) or (Insufflation[Title]) or (laparo*) or (robot*)) and (("Mixed gas") or ("ideal gas") or (nitrogen) or ("Gas mixture"))		523
EMBASE	1. (pneumoperitoneum or insufflation or laparo* or robot*).mp 2. ("mixed gas" or "ideal gas" or "gas mixture").mp 3. 1 and 2		95 (dupe with Medline
MEDLINE	1. (pneumoperitoneum or insufflation or laparo* or robot*).mp 2. ("mixed gas" or "ideal gas" or "gas mixture").mp 3. 1 and 2		95 (dupe with embase)
Clinicaltrials.gov	Pneumoperitoneum OR insufflation OR robotic surgery	With results	122

### Eligibility criteria

Due to the limited number of human and animal studies, it was deemed appropriate to include both types of studies. Table 2 shows the criteria for studies to be included/excluded.

**Table 2:** Shows the inclusion criteria and exclusion criteria for the studies identified in the database search

Inclusion criteria	Exclusion criteria
Laparoscopic abdominal and gynaecological pelvic surgeries	Secondary sources such as reviews, newsletters, government, and legal information
Insufflation at standard pressure (12mmg to 16 mmHg)	Novel single gas type studies, e.g., reactive oxygen scavengers
Non-randomised studies and RCT studies	Trials utilising mixed gas mixtures with no obtainable results
Studies utilising gas mixtures	Studies where no English translation was available
Animal and human studies	
CO_2_ insufflation as the comparison group	Intervention arms or experiments comparing the utilisation of air as an experimental gas mixture

### Study selection

From the database search, studies were collated using Endnote 20. Any duplicates were removed, and each title and abstract were screened for eligibility. Full-text articles were retrieved if the eligibility criteria were unclear based on abstract screening. Full texts of potentially relevant articles were screened and assessed against the eligibility criteria thereafter. Any discrepancies were discussed between the two reviewers.

### Data extraction

Two reviewers undertook the process of data extraction. The study data extracted from the processed articles included the author, year of publication, country of study, design study, sample size, intervention received, procedure performed and their results. This was organised by human and animal studies. Any studies missing data or had unobtainable results were excluded from the review. Due to the high heterogeneity noted in the study design, subject population and outcomes assessed, a narrative synthesis was agreed to be the most appropriate approach.

### Quality assessment of individual studies

The ‘Risk of Bias in Non-randomised Studies—of Interventions’ (ROBINS-I) tool [10] was used to assess the level of bias in papers utilising non-randomised methods of intervention. For RCT, the Cochrane “Risk of Bias 2’ (RoB 2) [11] tool was utilised. An initial assessment of bias was completed by one reviewer, and a discrepancy check was then performed by a second reviewer.

## Results

**Fig. 1:**
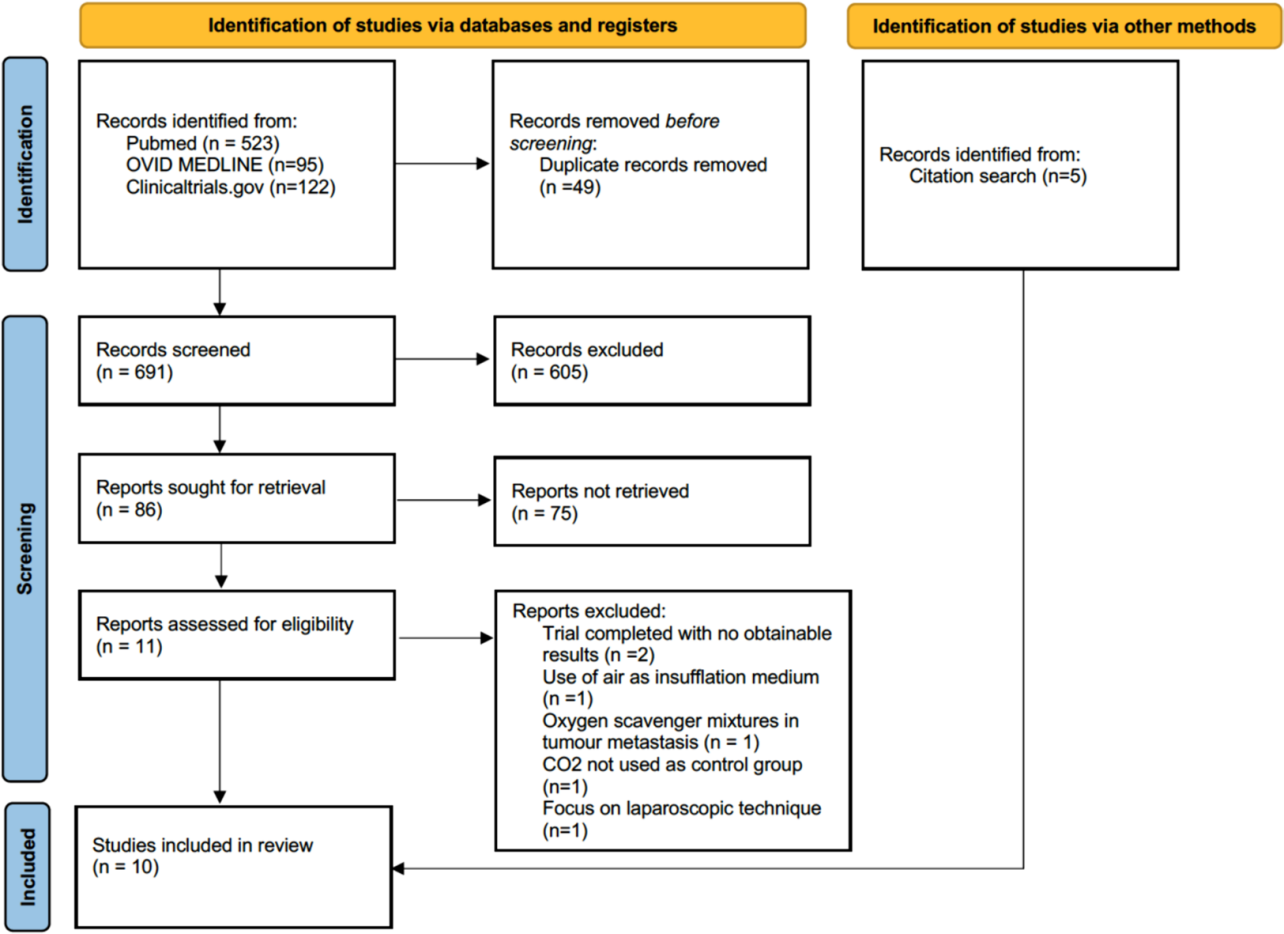
Systematic literature search summarised using the PRISMA 2020 flow diagram

A total of 691 articles were found after the removal of duplicates in the initial systematic literature search. Of these 86 articles were sought for full-text screening. A further 5 articles were found through citation search totalling 10 articles included in the review as shown in Fig. 1**.** Dates of publications range from 2002 to 2021 with all studies completed in Europe [3, 12–20]. We expected to find studies from outside of Europe, however, due to the little literature present, this was not possible. Further study characteristics are documented in Table 3.

**Table 3:** (13–22) Study characteristics of all studies included in the review

Author	Animal or human	Country	Study design	Gas mixture intervention and insufflation pressure (mmHg)		n	Outcome measure	Conclusions of the primary outcome
[3] Wildbrett 2002	Animal (Inbred BD IX rats)	Germany	Non-randomised intervention	Group 1: CO_2_ Group 2: CO_2_ + 20% O_2_	10 10	10 10	Tissue oxygen partial pressure (mmHg)	CO_2_ insufflation caused a significant decrease in tissue oxygen partial pressure from 23 to 5 mmHg (*p* = 0.000). Insufflation with a gas mixture of 80% CO_2_ and 20% O_2_ resulted in no significant difference between the control and intervention (*p* = 0.494)
[12] Elkelani 2004	Animal (Female mice)	Belgium	Randomised control trial	Group 1: CO_2_ Group 2: CO_2_ + 3% O_2_ Group 3: CO_2_ + 6% O_2_ Group 4: CO_2_ + 9% O_2_ Group 5: CO_2_ + 12% O_2_	15 15 15 15 15	5 5 5 5 5	Adhesion score Adhesion proportion	Adhesions decreased after the addition of 3% oxygen (*P* = 0.05). Compared to CO_2_ + 3% O_2_, adhesions increased with the addition of 6% (*P* = 0.05), 9% (*P* = 0.01) and 12% (*P* = 0.02) oxygen
[13] Binda 2009	Animal (Female BALB/c mice)	Belgium	Randomised control trial	Group 1: CO_2_ Group 2: CO_2_ + 3% O_2_	15 15	4 4	Adhesion score Adhesion proportion	Adhesions were reduced by adding 3% oxygen to the pneumoperitoneum (*P* = 0.05)
[14] Corona 2011	Animal (Female BALB/c mice)	Belgium	Randomised control trial	Group 1: CO_2_ + 4% O_2_ Group 2: CO_2_	15 15	4 4	Adhesion score Adhesion proportion (results unobtainable) Inflammation score	In comparison with the control group (group 1), both the total and proportion of adhesions increased when a pure CO_2_ pneumoperitoneum was used (group 2; *P* = 0.007 and *P* = 0.0053, respectively)
[15] Corona 2013	Animal (Female BALB/c mice)	Belgium	Randomised control trial	Group 1: CO_2_ Group 2: CO_2_ + 5% N2O Group 3: CO_2_ + 10% N_2_O Group 4: CO_2_ + 25% N_2_O Group 5: CO_2_ + 50% N_2_O	15 15 15 15 15	5 5 5 5 5	Adhesion proportion Adhesion score (results unobtainable)	Varying percentages of N_2_O decreased the total and proportion of adhesions in all groups (P = 0.0001 and P = 0.0001, respectively). Adhesion formation between varying N_2_O percentages were not significantly different
[16] Binda 2014	Animal (Female BALB/cJRj mice)	Belgium	Randomised control trial	Group 1: CO_2_ Group 2: CO_2_ + 10% N_2_O^a^ Group 3: CO_2_ + 10% N_2_O + 4% O_2_^a^	15 15 15	6 6 6	Total tumours (1 week after tumour implantation in mice before intervention)	Intermediary steps failed to reach significance; that is CO_2_ + 10% N_2_O (1 vs 2), and CO_2_ + 10% of N_2_O + 4% of O_2_ (1 vs 3)
[17] Binda 2021	Animal (Female BALB/cJRj mice)	Belgium	Randomised control trial	Group 1: no intervention Group 2: CO_2_ Group 3: CO_2_ + 4% O_2_	NA 15 15	8 8 8	The effect on peritoneum morphology was assessed by scanning electron microscopy (SEM)	In samples taken 24 h after surgery, an increased cell retraction (*p* = 0.005), and changes in the total score (*p* = 0.03) were observed in the group 60 min of hypoxia with humidified CO_2_. The effect of adding O_2_ on mesothelial morphology did not reach significance
[18] Koninckx 2013	Human Female	Belgium	Randomised control trial	Group 1: CO_2_ Group 2: CO_2_ + 10% N_2_O + 4% O_2_^a^	15 15	11 16	Adhesion score VAS score	In the control group, a significantly high number of adhesions were found in all women. 1 filmy adhesion was found in 4 women of the full conditioning group; 12/16 were adhesion free. (*P* = 0.0005)
[19] Verguts 2015	Human Female	Belgium	Randomised control trial	Group 1: CO_2_ + Ringer’s lactate Group 2: CO_2_ + Icodextrin 4% Group 3: CO_2_ + 10% N_2_O + 4% O_2_ + Ringer’s lactate^a^ Group 4: CO_2_ + 10% N_2_O + 4% O_2_ + Icodextrin 4%^a^	15 15 15 15	5 5 5 5	Resorption rates of fluids used in the prevention of adhesion after laparoscopic surgery	Group 3 showed a decreased resorption rate of Ringer’s lactate after 24, 48, and 72 h (*P* = 0.03, 0.009, and NS vs group 1), respectively. Group 4 did not affect the resorption rate of Icodextrin (NS vs group 2)
20 Storme 2016	Human Female	Belgium	Randomised control trial	Group 1: CO_2_ Group 2: CO_2_ + 10% N_2_O + 4% O_2_^a^	15 15	7 7	Adhesion score VAS score	A significantly high number of adhesions were found in all control patients. In the limited conditioning group, filmy adhesions were found in 4/7 women, while the remainder were adhesion free. Discontinuation of the surgeon, resulted in only 14/40 patients receiving surgery resulting in a reduction of power (*P* = 0.2040)

Gas mixture interventions with ^a^ include other factors are mentioned in the discussion section. Insufflation pressures of gases have also been included

VAS score: Visual Analogue Scale for pain scoring

## Summary of the reviewed literature

### Adhesion score

Three randomised animal studies comprising 41 female BALB/c mice, utilised different gas mixtures to evaluate adhesion score [12–14]. Only the use of CO_2_ + O_2_ gas mixtures were used to form the insufflation medium in these studies. Where O_2_ concentrations < 5% were added to the CO_2_, adhesion formation decreased compared to standard laparoscopy, whilst concentrations > 5% as tested in one study [12], resulted in increased adhesion score compared to 3% [3% (3.1 ± 4) vs 6% (5.3 ± 4 NS) vs 9% (6.4 ± 1.5 *P* = 0.01) vs 12% (5.7 ± 5 NS)].

Two randomised human studies comprising 41 female patients undergoing laparoscopic surgeries also evaluated adhesion score as its outcome [18, 20]. Both studies utilised conditioning, which uses CO_2_ + 10% N_2_O + 4% O_2_. However, in one study [18] full conditioning was utilised in the intervention group which includes, cooling of the pneumoperitoneum to 31c, humidification, and administration of dexamethasone post-operatively. In the other study [20] only limited conditioning was utilised, which does not involve humidification and cooling. Both studies saw a reduction in the number of adhesions and all patients in the intervention groups either had filmy adhesions or were adhesion free. The study utilising limited conditioning failed to reach statistical significance, which was due to a small sample size as the surgeon moved facilities before the trial was over (adhesion score: CO_2_ (155 ± 119) vs limited conditioning (95 ± 99 *P* = 0.204)).

### Adhesion proportion (%)

Three randomised animal studies comprising 58 female BALB/c mice measured the adhesion proportion [12, 13, 15]. Two studies utilised CO_2_ + O_2_ gas mixtures whilst the other study tested differing concentrations of N_2_O with CO_2_. For studies where O_2_ concentrations were < 5% [12, 13], adhesion proportion decreased compared to pure CO_2_ (adhesion proportion: CO_2_ (42 ± 25) vs 3% O_2_ (20 ± 20 *P* = 0.02) and CO_2_ (34.4 ± 17.5) vs 3% O_2_ (23.4 ± 13.8 *P* = 0.05), respectively). Whilst O_2_ concentrations > 5%, resulted in increased adhesion proportions compared to 3% (3% (20 ± 20) vs 6% (43 ± 45 *P* = 0.05) vs 9% (46 ± 20 *P* = 0.01) vs 12% (51 ± 30 *P* = 0.02)). In the third animal study [15], differing concentrations of N_2_O are mixed with CO_2_ and the adhesion proportion is measured as the outcome. With increasing concentrations of N_2_O, adhesion formation decreases, and this can be seen from as little as 5% addition of N_2_O (CO_2_ (24 ± 7.5) vs 5% (6.5 ± 5 *P* = 0.001) vs 10% (2 ± 2.5 *P* = 0.001) vs 25% (4 ± 1.25 *P *= 0.001) vs 50% (1 ± 0.5 *P* = 0.001)).

### Tissue partial pressure

One non-randomised interventional study, comprising 20 inbred rats, evaluated the effect of adding 20% O_2_ to the CO_2_ pneumoperitoneum, with monitoring of tissue partial pressure as an outcome measure [3]. Insufflation with the non-hypoxic gas mixture resulted in similar partial pressures as to using air and caused no significant difference (*P* = 0.494) whilst the use of CO_2_ insufflation resulted in a significant decrease of partial pressures of O_2_ from 23 to 15 mmHg (*p* = 0.000).

### Tumour implantation around the surgical site

One randomised animal study [16] using female BALB/cJRj mice, utilised gas mixtures of CO_2_ + 10% N_2_O + humidification and CO_2_ + 10% N_2_O + 4% O_2_ + humidification to evaluate the levels of tumour implantation around the surgical site by injection of tumour cells before laparoscopic surgery and dissection for results one week after surgery. CO_2_ insufflation increased tumour implantation in the abdominal cavity compared to no insufflation. Median and interquartile ranges were converted to mean and standard deviation [21]. (Total tumours: 30.6 ± 7.19 vs 9.75 ± 4.35 *P* = 0.026.) The interventional groups failed to reach significance. (CO_2_ (30.6 ± 7.19) vs CO_2_ + 10% N_2_O (18.875 ± 8.23 NS) vs CO_2_ + 10% N_2_O + 4% O_2_ (23 ± 9.7 NS).)

### Resorption rate of fluid barriers

One randomised human study, comprising 20 female patients, utilised CO_2_ + 10% N_2_O + 4% O_2_ (as well as humidification and cooling) + Ringer’s lactate/icodextrin 4% [19]. This study compared the resorption rate of fluid barriers that prevent adhesion formation. The resorption rate of both fluid barriers was fast and slowed down as the volume dropped, where CO_2_ + ringer’s lactate after 24, 48, 72 h was 11.7% ± 1%, 38.2% ± 9.5%, 5.3% ± 0.6%, respectively. The use of the gas mixture + Ringer’s lactate saw a decrease in resorption rate and after 24, 48, 72 h, 25.2% ± 3.4%, 13.2% ± 1.3%, and 6% ± 0.5%, respectively (*P* = 0.03, 0.009, and NS). However, the use of a gas mixture showed no significant difference in the resorption rate of icodextrin 4%.

### VAS score: Visual Analogue Scale score

Two randomised human studies comprising 41 female patients, also evaluated VAS score, which is the measure of pain, as an outcome measure. The respondent rates their level of pain on a line from “no pain” to “worst pain possible”. Both studies saw a reduction in reported VAS scores from the interventional group compared to the use of 100% CO_2_. In one of the studies [18], postoperative pain scores after day 1 were significantly reduced in the interventional group (VAS score for day 1: CO_2_ (4 ± 2.6) vs CO_2_ + 10% N_2_O + 4% O_2_ (1.2 ± 1)). For the second study [20], VAS scores postoperatively were lower in the interventional group during all days measured and significantly reduced from day 1 (VAS score for day 1: CO_2_ (4 ± 1.3) vs CO_2_ + 10% N_2_O + 4% O_2_ (2.4 ± 1.4 *P* = 0.0405).

### Risk of bias

Risk of bias assessment overall showed no concerning levels of bias. This is presented as a table in Supplementary Materials 1,2. Of the 10 studies included, 7 had a low overall risk of bias, whilst the other 3 studies had a moderate risk of bias. Predominant concerns were largely due to deviation from intended interventions. This was due to 2 mice dying during intubation (Corona 2013), 6 dropouts (Koninckx 2013), and the surgeon changing facilities (Storme 2016). Koninckx’s (2013) paper also showed some moderate risk of bias in the randomisation process. There seemed to be a significantly greater adhesion score pre-operatively in the control group compared to the intervention group. Verguts’ (2015) paper also showed a risk of bias due to the method of outcome measure. It is mentioned in this paper that the method of measuring is inaccurate and may affect the results.

## Discussion

To date, extensive literature has been published on the use of alternative gases for insufflation, with a meta-analysis of these studies and their outcomes published [22]. However, there has been little literature to support the use of mixed gases as an alternative to CO_2_ insufflation. Therefore, this systematic review and subsequent narrative synthesis aims to evaluate the efficacy of various gas mixtures and concentrations to highlight potential alternatives to the use of CO_2_ pneumoperitoneum in minimally invasive surgery.

The purpose of the preliminary animal studies included in this review were to identify therapeutic concentrations of different gas mixtures. Mice studies in our review have highlighted that adhesion formation significantly decreased with O_2_ concentrations below 5%, whilst concentrations greater than 5% resulted in a similar, if not greater increase in adhesion formation [12, 13]. Another preliminary animal study [15] reported that the addition of N_2_O to the CO_2_ pneumoperitoneum was effective in preventing adhesions in a dose respondent curve and continued to do so until 100% N_2_O was reached, although this decrease in adhesion formation was exponentially limited. In Koninckx’s (2013) pilot study a combination of O_2_, N_2_O and CO_2_ was used to insufflate the abdomen during laparoscopic intervention, which resulted in a significant decrease in adhesion scores and proportion [18]. This combination of CO_2_ + 4% O_2_ + 10% N_2_O was named “conditioning” of the pneumoperitoneum. However, when utilising the same gas mixture in the paper published by Storme (2016), reduction in adhesion score failed to reach significance [20]. The author attributed this to the trial being prematurely terminated due to the discontinuation of the surgeon. Whether this validates the finding that “conditioning” significantly decreases adhesions post-operatively in human studies is hard to determine. Despite this, there was still a significant reduction in post-operative pain outcomes for both studies [18, 20].

The therapeutic concentrations of less than 5% O_2_ can be attributed to the pO_2_ of the pneumoperitoneum when using this gas mixture. With CO_2_ + 3% O_2_, a pO_2_ of 28 mmHg was measured, which is comparable to the intracellular pO2 that is normally found in the peritoneal tissue [12, 23] whilst with high O_2_ concentrations, there is an increase in tissue partial pressure and a subsequent increase of reactive oxygen species (ROS)[24]. Prior research has concluded that ROS are deleterious for cells and their release leads to inflammation and activation of the coagulation cascade, subsequently increasing adhesion formation. [25, 26]. Regarding N_2_O, this gas has typically been avoided due to its explosion risk. Previous case reports highlighted that using diathermy whilst inflating with pure N_2_O resulted in the combustion of trapped methane gas and the death of patients [27, 28]. However, concentrations below 29% are safe for use with diathermy [29], and in Corona’s (2013) study, they demonstrated that as little as 5% N_2_O produced similar results in reducing postoperative adhesions compared to pure N_2_O.

Other findings that were made during this review included outcomes of tumour implantation rates and resorption rates of different fluid adhesion barriers. Currently, the reported incidence varies from 1 to 19.65% depending on the primary malignancy and stage of disease [30]. A previous study in dogs suggested that in the presence of CO_2_, peritoneal pH levels dropped during insufflation with CO_2_ [31]. Binda’s (2021) paper was able to show these morphological changes and concluded that this drop in pH resulted in a reduction of clotting time and an increase in turbidity of the fibrin clot around the site of trauma. This in turn led to increased resistance of metastatic cells against macrophages within the peritoneum and suggests why port site metastasis is more likely to occur in a hypercapnic peritoneal environment [3]. However, in Binda’s (2014) trial, tumour implantation rates did not significantly decrease when comparing “conditioning” to the use of pure CO_2_ in mouse models, which the author suggested may be due to the lack of size in the sample [16].

Verguts’ (2015) study centred their observations around the peritoneal resorption rate of fluid adhesion barriers whilst utilising “conditioning”. Using these barriers and preventing contact between two points of surgical trauma reduces the number of adhesions as mediators of adhesion formation are present up to 7 days after surgery [32]. Current barriers include Ringer’s lactate and Icodextrin 4%. Currently, there are many alternatives to these cited barriers including composite polymers such as Prevadh™, which has shown a significant reduction in pelvic adhesions and severity compared to Ringer’s lactate solution [33]. This study finds a significant decrease in the resorption rate of Ringer’s lactate, increasing the time that this barrier is present in the peritoneal space, however, does not show a significant difference in the resorption rate of icodextrin 4%.

Confounding factors affecting adhesion formation were considered for each paper, including bowel handling and inflammation. Upon reviewing the articles involving animal research, we concluded that this heterogeneity was mitigated within studies. Each publication provided a thorough explanation of the standardised adhesion-forming process and many of these studies produced ten 1.6-mm lesions. Furthermore, the duration of pneumoperitoneum was also controlled, subsequently reducing variability in levels of inflammation due to pneumoperitoneum exposure of which the morphological effects are discussed in Binda’s Paper [17]. Select studies [12–14] highlighted that only one surgeon operated on subjects. Unfortunately, many of the other animal studies did not disclose the number of surgeons within trials, which remains a limitation of our paper as this suggests that there may have been some variation in the bowel manipulation techniques used. When reviewing pilot human studies, each paper had only one surgeon and one form of surgical procedure to minimise variability.

Several of these papers lack the necessary sample sizes to develop concrete conclusions concerning the efficacy of mixed gases, which is a limitation of our narrative synthesis and justification for not completing a meta-analysis. There are also concerns that some of these studies include multiple additional factors such as cooling and humidifying the gas as part of their intervention. As mentioned in the results section, there is some discrepancy between full and limited “conditioning” and a letter to Koninckx's (2013) translational study also touched on this [34]. The letter highlighted that it is challenging to distinguish the weighting and efficacy of each factor involved in adhesion prevention. Furthermore, if adverse effects were to occur, the identification of the responsible factor may become problematic. Another limitation may be the capabilities of different insufflation devices; many of which are not designed to insufflate with multiple gases such as the Airseal ® iFS or ENDOFLATOR® 50 [35]. If pre-developed gas mixtures were introduced into the system, issues such as turbulent flow and ineffective smoke evacuation may develop, both of which are problematic due to current COVID-19 guidelines and the increased awareness of the harms of surgical smoke [36, 37].

More evidence is required to develop a clearer understanding of the efficacy of mixed gases. This includes examining a wider range of clinical outcomes such as the risk of embolisms, cardiorespiratory problems, and postoperative survival and infection rates. Adhesion formation and risk of tumour implantation should also be revisited with larger sample sizes completed. Though this is the case, studies mentioned in this review showcase promising results that suggest mixed gas pneumoperitoneum in minimally invasive surgery may be viable in the future.

## Conclusion

This review summarises the results of various papers relating to the utilisation of mixed gas insufflation in laparoscopic surgery and has several implications for laparoscopic surgery. The combination of CO_2_ + 10%N_2_O + 4%O_2_ seemingly provides benefits that are favoured over the use of pure CO_2_ and when trialled in humans, show a significant reduction in adhesion formation, VAS score and inflammatory response. Nonetheless, further research with larger sample sizes must be completed to evaluate other clinical outcomes and physiological parameters including blood gas levels, pH levels and metabolite values, both in animal studies and translational human studies. Currently, several of the studies available are seemingly underpowered and require larger sample sizes in more studies to develop a clearer understanding on the effects of different gas mixtures. Furthermore, the number of additional confounding factors in RCT should be reduced so that each component of the current suggested gas mixture and conditioning procedure can be extensively tested for safety and efficacy.

## Supplementary Information

Below is the link to the electronic supplementary material.

Supplementary file1 (DOCX 17 KB)

## Data Availability

Data supporting this study are included within the article and/or supporting materials.
