# Supplementary material for: Use of mixed gas pneumoperitoneum during minimally invasive surgery: a systematic review of human and mouse modelled laparoscopic interventions
Source: J Robot Surg. 2024 May 17;18(1):215. doi: 10.1007/s11701-024-01971-1 (PMC11101563; doi:10.1007/s11701-024-01971-1)
Supplement: Supplementary file 1 — Supplementary file1 (DOCX 17 KB) [file 11701_2024_1971_MOESM1_ESM.docx]

Use of mixed gas pneumoperitoneum during minimally invasive surgery. A systematic review of human and mouse modelled laparoscopic interventions.

Leon Chen^1^, Prokar Dasgupta ^2,3^, Nikhil Vasdev ^4,5^

1. Faculty of Life Sciences & Medicine, King’s College London, London, UK
2. Department of Urology, Guy’s & St Thomas’ NHS Foundation Trust, London, United Kingdom
3. MRC Centre for Transplantation, King’s College London, London, United Kingdom
4. Hertfordshire and Bedfordshire Urological Cancer Centre, Department of Urology, Lister Hospital, East and North Hertfordshire NHS Trust, Stevenage, UK
5. School of Life and Medical Sciences, University of Hertfordshire, Hatfield, UK

Email of corresponding author: [Leon.chen@kcl.ac.uk](mailto:Leon.chen@kcl.ac.uk)

# Supplementary Material

## S1: Risk of Bias utilising the Cochrane ROB 2 tool for randomised trials

| Randomised Trials – Cochrane RoB 2 tool | | | | | | |
| --- | --- | --- | --- | --- | --- | --- |
| Author | Randomisation Process | Deviation from Intended Intervention | Missing Outcome Data | Measurement of the Outcome | Selection of the Reported Result | Overall Judgement |
| Elkelani 2004 | Low | Low | Low | Low | Low | Low |
| Binda 2009 | Low | Low | Low | Low | Low | Low |
| Corona 2011 | Low | Low | Low | Low | Low | Low |
| Corona 2013 | Low | Some Concerns | Low | Low | Low | Low |
| Binda 2014 | Low | Low | Low | Low | Low | Low |
| Binda 2021 | Low | Low | Low | Low | Low | Low |
| Koninckx 2013 | Some Concerns | Some Concerns | Low | Low | Low | Some Concerns |
| Verguts 2015 | Low | Low | Low | Some Concerns | Low | Some Concerns |
| Storme 2016 | Low | Some Concerns | Low | Low | Low | Some Concerns |

## S2: Risk of Bias utilising the ROBINS-I tool for non-randomised trials

| Non-randomised Trials – ROBINS-I tool | | | | | | | | |
| --- | --- | --- | --- | --- | --- | --- | --- | --- |
| Author | Confounding | Selection | Classification of Intervention | Deviation from Intended Intervention | Missing Outcome Data | Measurement of Outcome | Reported Result | Overall Judgement |
| Wildbrett 2002 | Low | Low | Low | Low | Low | Low | Low | Low |
